# Supermarket Transaction Records In Dietary Evaluation: the STRIDE study: validation against self-reported dietary intake

**DOI:** 10.1017/S1368980023001842

**Published:** 2023-12

**Authors:** Victoria Jenneson, Darren C Greenwood, Graham P Clarke, Tim Rains, Bethan Tempest, Becky Shute, Michelle A Morris

**Affiliations:** 1 Leeds Institute for Data Analytics, Level 11 Worsley Building, Clarendon Way, University of Leeds, Leeds LS2 9JT, UK; 2 School of Geography, Seminary St, Woodhouse, University of Leeds, Leeds LS2 9JT, UK; 3 Leeds Institute of Cardiovascular and Metabolic Medicine, University of Leeds, Woodhouse, Leeds LS2 9JT, UK; 4 Sainsbury’s Plc, 33 Holborn, London EC1n 2HT, UK; 5 Leeds Institute of Medical Research, St James’s University Hospital, University of Leeds, Beckett St, Harehills, Leeds LS9 7TF, UK

**Keywords:** Dietary assessment, Validation, Macronutrients, Supermarket, Public health, Methods, Transaction data

## Abstract

**Objective:**

Scalable methods are required for population dietary monitoring. The Supermarket Transaction Records In Dietary Evaluation (STRIDE) study compares dietary estimates from supermarket transactions with an online FFQ.

**Design:**

Participants were recruited in four waves, accounting for seasonal dietary variation. Purchases were collected for 1 year during and 1 year prior to the study. Bland–Altman agreement and limits of agreement (LoA) were calculated for energy, sugar, fat, saturated fat, protein and sodium (absolute and relative).

**Setting:**

This study was partnered with a large UK retailer.

**Participants:**

Totally, 1788 participants from four UK regions were recruited from the retailer’s loyalty card customer database, according to breadth and frequency of purchases. Six hundred and eighty-six participants were included for analysis.

**Results:**

The analysis sample were mostly female (72 %), with a mean age of 56 years (sd 13). The ratio of purchases to intakes varied depending on amounts purchased and consumed; purchases under-estimated intakes for smaller amounts on average, but over-estimated for larger amounts. For absolute measures, the LoA across households were wide, for example, for energy intake of 2000 kcal, purchases could under- or over-estimate intake by a factor of 5; values could be between 400 kcal and 10000 kcal. LoA for relative (energy-adjusted) estimates were smaller, for example, for 14 % of total energy from saturated fat, purchase estimates may be between 7 % and 27 %.

**Conclusions:**

Agreement between purchases and intake was highly variable, strongest for smaller loyal households and for relative values. For some customers, relative nutrient purchases are a reasonable proxy for dietary composition indicating utility in population-level dietary research.

National dietary surveys, such as the UK’s National Diet and Nutrition Survey (NDNS)^(1)^ can reveal dietary trends that impact health. However, costs and administrative burdens associated with surveys limit their sample sizes and temporal granularity due to their cross-sectional nature. Online food records such as myfood24^(2)^ and Intake24^(3)^ improve scalability and reduce costs associated with dietary surveys^(4)^. Yet, digital methods continue to rely on self-report which is known to exhibit social desirability and recall biases leading to under-estimation of energy intake^(5)^. Harnessing new technology could benefit research by providing a suite of scalable objective dietary assessment methods to complement existing self-report^(6)^. Objective dietary measures may come in the form of image capture techniques^(7)^, nutritional biomarkers^(7)^ and food system administrative data such as transaction records^(8)^.

Food purchases represent upstream dietary behaviours which precede consumption, and thus purchases represent a proportion of the total food available for household members to consume. Advancements in technology now permit the routine collection of purchase data by supermarkets in the form of Electronic Point of Sale (EPOS). EPOS data are used commercially for stock analysis, customer segmentation (e.g. regular purchases of nappies and infant formula would be indicative of a young family) and marketing. In combination with product nutritional information and customer data, EPOS transactions could provide objective population-level dietary insight at a much larger scale^(9)^. As such, researchers have begun to explore the value of supermarket electronic purchase records in a dietary research context^(8,10–13)^.

A precursor to digital purchase records was the collection of paper till receipts, often accompanied by purchase diaries. Paper receipts enable purchases from different sources to be combined, capturing total purchases more fully. Early work in the UK found paper till receipts demonstrated good statistical agreement with self-reported individual consumption^(14)^. Yet scalability of the method is limited by the need for participants to collect their receipts and the burden of manual coding by researchers. Furthermore, there remains the potential for participants to lose or systematically omit receipts.

Digitised receipt collection methods could benefit dietary assessment by eliminating these burdens and biases. Automatically captured electronic supermarket transaction records are becoming increasingly employed in dietary research and monitoring of dietary policy^(8)^, thanks to their scale, timeliness and richness of detail. However, the commercial nature of these data sources limits their capacity for linkage, meaning purchase data used in research typically represent a single retailer and are therefore unlikely to capture the entirety of the diet for most individuals. As a result, perfect agreement in absolute terms cannot be expected, yet there is a need to understand how well purchase data represent dietary behaviours in relative terms.

To date, only a few studies have investigated the validity of loyalty card purchase records as a dietary measure, highlighting the need for further validation studies. One such study by Eyles et al.^(10)^ included just forty-nine customers of a New Zealand supermarket and 3 months of transaction records. Comparison of relative nutrients from household transaction records with self-reported intake from four random 24-h dietary recalls, revealed differing strengths of correlation by nutrient, from weak (0·06 for Na) to moderate (0·54 for percentage energy from saturated fat). Similarly, in a study comparing grocery purchases with a FFQ for nearly 12 000 Finnish loyalty card holders, strength of association at the food group level varied substantially, with gamma statistics ranging from 0·12 for cooked vegetables to 0·75 for margarines^(12)^.

These previous validation studies point to the favourable potential of household supermarket transaction records to act as a proxy for individual dietary intake but suggest that the utility of the method is likely to depend on the food group or nutrient in question. Furthermore, while household composition and retailer loyalty appear to be important factors^(12)^, neither study attempted to account for household composition in their estimates, presenting an area for additional research. Comparisons with self-reported intake should also include alcohol, food waste and consumption by visitors to improve agreement^(10)^. Self-reported diet needs to cover more days, with larger sample sizes, to allow for large-intra-individual variation, particularly for sugar and total energy^(12)^.

This paper presents results from the Supermarket Transaction Records In Dietary Evaluation (STRIDE) study (protocol)^(15)^, which adds novel insight to the existing evidence by assessing the statistical agreement between estimates of nutrient purchases from loyalty card transaction records and estimates of nutrient intake from an online FFQ. The present study adds to existing knowledge by assessing statistical agreement and limits to agreement for both absolute and relative (energy-adjusted) macronutrient estimates, accounting for household composition to derive individual-level estimates of purchased nutrients.

## Methods

The study protocol^(15)^ was registered prior to starting the study on the Open Science Framework and is available online at ·https://doi.org/10·17605/OSF.IO/VUKTQ.esrc

### Study design

**Fig. 1:**
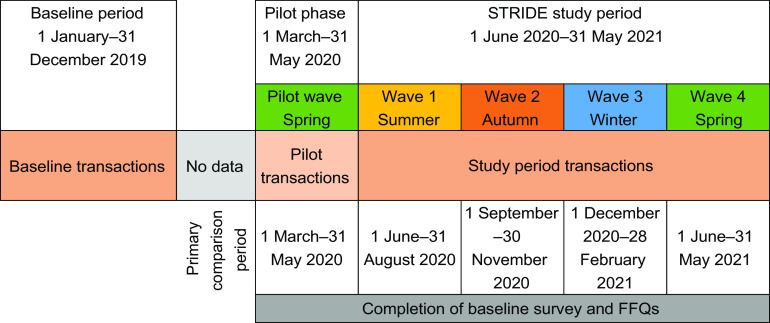
STRIDE study design. STRIDE, Supermarket Transaction Records In Dietary Evaluation

This validation study compares self-reported intake against household food purchase data from a major UK retailer’s loyalty card scheme. Intake is captured for the previous 2–3 months using an online FFQ by the Scottish Collaborative Group (SCG)^(16)^. The study recruited four waves of study participants (plus a pilot wave) and was designed to capture intake and purchase data across all seasons. Transactions cover a 1-year baseline period prior to study recruitment and a 1-year period during which the STRIDE study took place. Transaction data which cover the same 3 months as that which is captured by the FFQ for each wave, is referred to as the ‘primary comparison period’. The period covered by each wave is depicted in Fig. 1. More detail on the participant recruitment is provided below.

### Participant sampling and recruitment

Participants were sampled from the retailer’s database of loyalty card holders. Customers must be at least 18 years old to hold a loyalty card. Eligible customers were required to have an email address on file, to have opted in to receive research communications and to have their loyalty card registered to an address in one of four regions in England (Yorkshire and the Humber, South-East, East Midlands, and West Midlands), selected to cover a range of geographic and demographic characteristics. Primary shoppers were selected, for whom we considered purchases at the study retailer likely to represent the majority of their shopping. This was determined by selecting only those customers who shopped in at least seven out of fifteen food categories on a minimum of ten occasions during the 2019 calendar year, as in the study by Clark et al.^(17)^. Additionally, customers with an annual spend on food and non-alcoholic beverages greater than 1·5 times the interquartile range beyond the upper quartile of annual spend published in the 2019 edition of the Family Food Survey (FFS)^(18)^ were excluded. The same proportion was also excluded from the lower end of the distribution of annual spend.

**Fig. 2:**
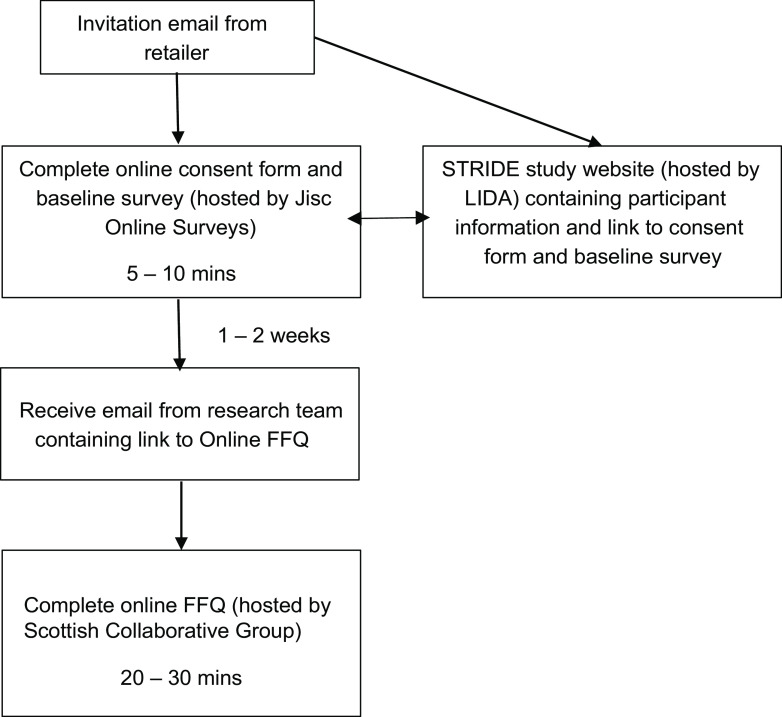
STRIDE participant journey flow diagram. STRIDE, Supermarket Transaction Records In Dietary Evaluation. LIDA, Leeds Institute for Data Analytics

A pilot wave was used to determine the expected sign-up and completion rates for the study and to test the flow of the participant journey. This indicated a sign-up rate of about 1 % and a completion rate for the FFQ of about 50 %. To achieve a sample size of 200 customers per wave for statistical agreement testing, all customers who met the eligibility criteria (about 45 000) were invited by the retailer via email to take part in one of the STRIDE study waves. The participant journey is shown in Fig. 2. Customers received an invitation email containing two links, one to the online consent form and baseline questionnaire hosted by Jisc Online Surveys, and the other to the study website (hosted by the Leeds Institute for Data Analytics (LIDA)) where participant information could be found.

Customers consented to receive a further email containing a link to the SCG online FFQ, and to allow their loyalty card purchase records for 1 year prior to, and 1 year during the study, to be shared with the research team. Customers provided their loyalty card number to enable their purchase records to be identified by the retailer. A unique customer identifier was also embedded into the URL in the invitation email to aid identification in the event of typos in loyalty ID data entry by participants. Purchase records were linked to the FFQ and each participant’s baseline survey and dietary questionnaire (via a unique study ID assigned to each participant). Upon completion of the FFQ, customers were entered into a prize draw for a chance to win a £75 high street voucher (one per recruitment wave including the pilot wave) as an incentive to participate in the study.

### Data collection

Demographic information (date of birth, gender, ethnicity, and height and weight for calculation of BMI) were collected via an online baseline questionnaire. Participants additionally reported: the number and ages of other people in their household; the proportion of their food purchases made with the retailer by selecting one of five categories on the baseline questionnaire (0–20 %, 21–40 %, 41–60 %, 61–80 % or 81–100 %); dietary restrictions; food waste; and the impacts of the COVID-19 pandemic on their food purchase and consumption habits.

Food consumption data were collected via the SCG Online FFQ^(16)^, a 150-item semi-quantitative questionnaire which asks the participant to report the frequency and amounts consumed for each item. Transaction data and product nutrition information were provided by the retailer.

All food and beverages (including alcoholic beverages) purchased either in store or online with a scanned loyalty card were recorded. Transaction files contained a row for each product (with a unique product ID) with an item description, purchase quantity (units or weight as appropriate) and cost (GBP £). Products purchased on a single shopping trip may be linked by a transaction ID; thus, a transaction represents a basket of goods.

### Nutrient estimates

Daily nutrient intakes for each participant were estimated from their FFQ by the SCG team as part of their paid FFQ service, using the UK National Nutrient Databank^(16)^. Purchased nutrients were estimated from the transaction data by linking products to a bespoke product nutrient composition database via a unique product code (either the European Article Number (EAN) or Stock-keeping Unit (SKU)). The PNCD comprised of back of pack product nutrient information per 100 g or per 100 ml of product ((energy (kcal), total sugars (g), protein (g), total fat (g), saturated fat (g) and Na (mg)) provided by the retailer for products sold in 2019. This included retail own brand products and branded product information from Brandbank^(19)^. Seventy-two per cent of products were matched to product-specific nutrient data in the retailer file. For products where no specific match could be found in the product nutrient file, or where nutrition information was blank, generic nutrient values for the closest matching product were manually imputed from the UK’s Composition of Food Integrated Dataset (CoFID) version 7^(20)^. This was typically for non-packaged items such as fresh produce and in-store bakery items, alcohol (for which nutritional information is not legally required to be displayed on product packaging^(21)^) and seasonal products such as Easter eggs. After imputation, a match rate of 100 % was achieved.

The product weight was multiplied by the number of units purchased and its nutritional value per 100 g (or 100 ml; as specific gravity information was unavailable for products a simple approximation of 1 ml = 1 g was assumed) to derive the total nutrients purchased in a given period by each customer. For comparison with daily intake estimates, purchased nutrients were converted to mean daily household estimates by dividing the total nutrients purchased by the number of days in the primary comparison period (covering the same 3-month time frame as each FFQ). Individual-level daily purchase estimates were generated from household estimates by allocating purchased nutrients to individuals proportionate to UK dietary recommendations for energy intake by age and gender^(22)^ (Table 1). As genders were unknown for other household members, an average of recommended values for females and males was used. For example, if a study participant is a 30-year-old woman living with a 30-year-old partner and a 3-year-old child, she would be allocated 36 % of the nutrients purchased by the household (1928/(1928 + 2230 + 1197·5)) (Table 1).

**Table 1:** UK recommended daily energy intakes by age and gender source^(22)^

	Recommended daily energy intake (kcal)	
Age (years)	Female	Male
0–1	698	745
1–3	1165	1230
4–10	1656	1861
11–17	1959	2449
18–64	1928	2532
65+	1855	2215

Nutrient estimates are given both as absolute daily amounts and relative (energy-adjusted) values. Macronutrients are expressed in terms of their contribution to total energy by multiplying the number of calories per gram from the macronutrient (protein = 4 kcal/g, fat = 9 kcal/g, saturated fat = 9 kcal/g, sugars = 3·9 kcal/g) by the number of grams and then dividing by the total energy. This is then expressed as a percentage of total energy. Na is expressed in mg/kcal. Absolute estimates from purchase data are given at the household level and individual level, whilst relative purchase estimates are presented as a single figure. This is because the same proportions are used to allocate energy and all other nutrients to household members. Thus, the individual-level estimate would be the same as the household-level estimate.

### Statistical analysis

Daily nutrient purchase estimates (at the household level and at the individual level) for each primary comparison period are compared with individual-level nutrient intakes for the same time period. Purchased energy at the individual level is compared with energy intake (split by household size and self-reported customer loyalty). Due to low numbers, customers with a household size of three or more were combined and compared with single-person and two-person households. Similarly, customers reporting that the retailer contributes 0–20 %, 21–40 % or 41–60 % of their food purchases were combined to represent low-medium loyalty customers (0–60 % of food purchases) and compared with high loyalty customers (61–80 % of food purchases) and very high loyalty customers (81–100 % of food purchases). Individual-level purchase estimates for macronutrients and Na are compared with intake estimates.

Bland–Altman plots were generated to assess statistical agreement and limits of agreement (LoA)^(23,24)^. Bland–Altman is the gold standard method for comparison between methods used in clinical research. Plots show the mean of the two methods (x-axis) against the difference between the methods (y-axis). For the purposes of interpretation, agreement is expressed as the difference at a given magnitude of the mean, considering intake by FFQ as the reference point as it is the more established dietary assessment method. Neither method is perfect, and FFQ has itself been shown to under-estimate dietary intake.

Due to heteroskedasticity in the data (the difference between measures was related to the magnitude of the mean of the measures), values in the Bland–Altman plots are log-transformed. The axes of the Bland–Altman plots are back-transformed to aid interpretation and are shown as a ratio of purchase estimate/intake estimate against the mean^(24)^. This ratio may then be interpreted as a percentage difference. As the direction of the relationship was also dependent on the magnitude of measures, a regression approach was used to plot the mean difference (which is presented as a regression equation in the tables) and LoA, based on ±1·96 sd of the spread of residuals about the regression line^(24,25)^.

It was hypothesised that very low levels of purchasing at the supermarket are indicative of food being sourced from elsewhere (e.g. from other supermarkets or consumed at restaurants) rather than of low dietary intake. Therefore, a sensitivity analysis was performed to exclude customers purchasing less than 500 kcal/d (representing a quarter of an adult woman’s recommended intake). Statistical analyses were repeated on the sensitivity analysis sample.

## Results

### Participant characteristics

Recruitment figures for the STRIDE study are shown in Table 2. About half of the 1788 participants recruited across the whole study completed an online FFQ (*n* 825). Of those with completed FFQ records, 83 % (*n* 688) had made at least one purchase with the retailer in the corresponding 3-month period as covered by the FFQ. A further two participants were excluded as outliers; their estimated daily energy intake from the FFQ was ≥ 8000 kcal (four times the recommended energy intake for an adult woman). Results presented are pooled across all waves (including the pilot) for those 686 participants.

**Table 2:** STRIDE participant recruitment summary

Recruitment wave	Pilot	%	Wave 1	%	Wave 2	%	Wave 3	%	Wave 4	%	All waves combined	%
Number of participants consented	80		377		547		430		354		1788	
Number of participants with completed FFQ (%)	38	48	190	50	235	43	192	44	170	48	825	46
Analysis sample. (number of participants with completed FFQ and purchase data in the primary comparison period (%))	13	16	159	42	201	37	159	37	156	44	688	38

STRIDE, Supermarket Transaction Records In Dietary Evaluation.

The demographic characteristics of study participants are shown in Table 3. The majority of participants were female (72 %) and from a White ethnic background (97 %). Participants had a mean age of 56·2 years (sd 12·9 years) and an average household size of 2·2 persons. According to their self-reported height and weight, 54 % of participants were classified as overweight or obese. Thirty per cent had a loyalty card registered to an address in the Yorkshire and Humber region, 20 % in the East Midlands, 18 % in the West Midlands and 30 % in the South East. Participants were relatively affluent overall with almost 69 % living in areas in the five least deprived deciles according to the Index of Multiple Deprivation (IMD)^(26)^, and the most commonly inhabited Output Area Classification Supergroup areas^(27)^ were Suburbanites (31 %), Urbanites (27 %) and Rural Residents (17 %). Participants were also relatively loyal to the study retailer, with 82 % reporting to purchase more than 40 % of their food and beverages with the retailer and 64 % purchasing more than 60 %.

**Table 3:** STRIDE participant characteristics for all waves (including pilot) combined

Participant characteristics	Number of consented participants (*n* 1788)		Number of participants with completed FFQ (*n* 825)		Analysis sample (number of participants with completed FFQ and purchases recorded in the primary comparison period) (*n* 686)	
	*n*	%	*n*	%	*n*	%
Gender (%)						
Female	1303	73	597	72	497	72
Male	479	27	224	27	186	27
Other/unknown	6	0	4	1	3	0
Age						
Mean	56		57		56	
sd	14		13		13	
Ethnicity (%)						
White	1711	96	803	97	667	97
Non-White	52	3	12	2	9	1
Other/unknown	25	1	10	1	10	1
BMI (kg/m^2^)						
Underweight	187	11	91	11	73	11
Healthy	496	28	249	30	215	31
Overweight	513	29	241	29	198	29
Obese	382	21	163	20	135	20
Morbidly obese	112	6	50	6	38	6
Government Office Region						
Yorkshire and The Humber	519	29	249	30	209	31
East Midlands	338	19	161	20	135	20
West Midlands	360	20	146	18	126	18
South East	484	27	233	28	206	30
Other/unknown	87	5	36	4	10	2
IMD decile						
1 – most deprived	73	4	31	4	27	4
2	96	5	31	4	25	4
3	114	6	46	6	38	6
4	136	8	59	7	49	7
5	183	10	85	10	74	11
6	188	11	106	13	85	12
7	202	11	91	11	76	11
8	221	12	103	13	95	14
9	223	13	96	12	85	12
10 – least deprived	281	16	150	18	130	19
Unknown	71	4	27	3	2	0
Output Area Classification (OAC) Supergroup (2011)						
Rural Residents	254	14	142	17	116	17
Cosmopolitans	45	3	27	3	23	3
Ethnicity Central	15	1	5	1	4	1
Multicultural Metropolitans	124	7	55	7	46	7
Urbanites	465	26	208	25	183	27
Suburbanites	522	29	249	30	216	32
Constrained City Dwellers	75	4	20	2	16	2
Hard-pressed Living	217	12	92	11	80	12
Unknown	80	5	27	3	2	0
Share of purchases made with study retailer (%)						
0–20 %	122	7	58	7	44	6
21–40 %	239	13	97	12	81	12
41–60 %	309	17	145	18	119	17
61–80 %	456	26	195	24	174	25
81–100 %	661	37	330	40	268	39
Mean household size	2·3		2·2		2·2	
sd	1·3		1·1		1·1	

STRIDE, Supermarket Transaction Records In Dietary Evaluation; IMD, Index of Multiple Deprivation.

Overall, there was little difference in the characteristics of those who signed up for the study and those included in the analysis sample. Small observed differences include a smaller proportion of non-White participants, a higher proportion of healthy weight and a lower proportion of obese individuals, a smaller proportion of Constrained City Dwellers and a higher proportion of Rural Residents, as well as a slight reduction in household size in the analysis sample compared with total sign-ups.

### Descriptive statistics

Absolute daily estimates of purchased (household-level and individual-level) and consumed energy and nutrients are presented in Table 4. Household purchase estimates are about 80–90 % of the consumed estimate value, depending on the nutrient. Individual-level purchase estimates are about half the amount purchased at the household level. Individual-level purchase estimates are about 40 % of the estimated consumption amount.

**Table 4:** Absolute nutrient estimates from purchase records and FFQ (*n* 686)

	Absolute household purchase/day		Absolute individual-level purchase/day		Absolute consumption/day (FFQ)	
Nutrient	Median	IQR	Median	IQR	Median	IQR
Energy (kcal)	1746	803, 3233	910	371, 1621	1955	1584, 2480
Sugar (g)	82	35, 162	42	17, 83	107	83, 145
Protein (g)	65	27, 117	33	13, 60	83	65, 104
Total fat (g)	72	31 133	37	15, 66	79	61, 102
Saturated fat (g)	27	12, 52	14	6, 26	31	23, 41
Na (mg)	1984	781, 3661	1031	403, 1892	2623	2090, 3374

Relative (energy-adjusted) nutrient estimates are given for purchases and consumption in Table 5. Participants purchased on average 19 % of their energy from sugar, 14 % from protein, 36 % from fat, 14 % from saturated fat and an average of 1·06 mg Na per calorie.

**Table 5:** Energy-adjusted nutrient estimates from purchase records and FFQ (*n* 686)

	Energy-adjusted purchase/day		Energy-adjusted consumption/day (FFQ)	
Nutrient	Median	IQR	Median	IQR
Sugar (% energy)	19	16, 23	21	18, 25
Protein (% energy)	14	12, 16	17	15, 19
Total fat (% energy)	36	32, 41	37	33, 40
Saturated fat (% energy)	14	12, 16	14	12, 16
Na (mg/kcal)	1·1	0·9, 1·3	1·3	1·2, 1·5

### Agreement for absolute estimates

**Fig. 3:**
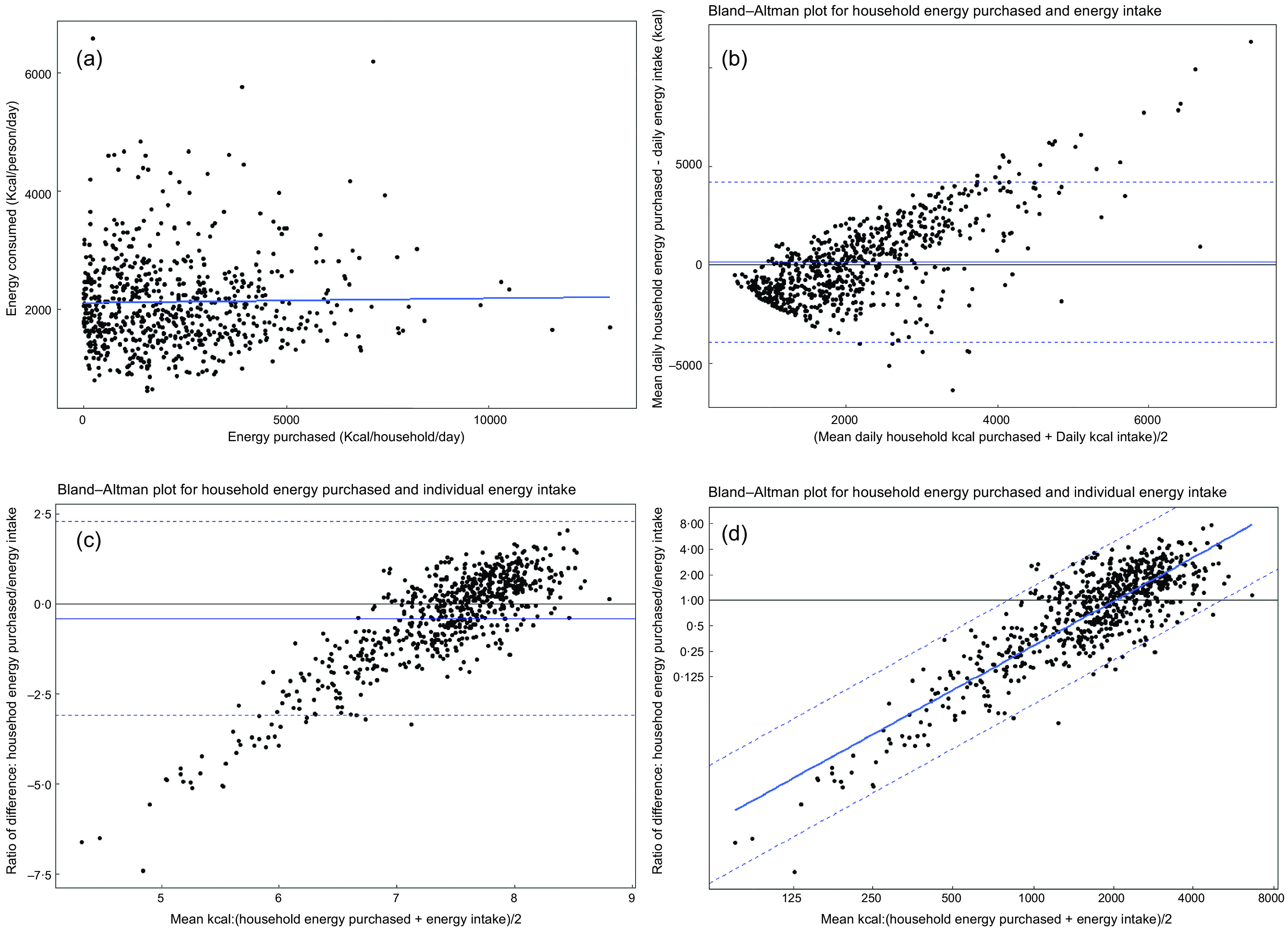
Household energy purchased *v* daily energy intake (kcal): (a) scatterplot, (b) Bland–Altman plot for agreement, (c) Bland–Altman plot for log-transformed variables, (d) Bland–Altman plot for log-transformed variables with regression approach, with difference expressed as a ratio of purchases:intake

The relationship between absolute daily purchases and absolute daily intake was examined for calories. As shown in the scatterplot in Fig. 3(a), correlation between the two measures is weak (Pearson’s correlation coefficient = 0·02). Correlation tells us to what extent measures follow the same linear pattern. This is not the same as agreement which tells us the average magnitude of difference between measures. Agreement between daily household energy purchased and daily energy intake by the participant can be seen in the Bland–Altman plot in Fig. 3(b), which plots the mean of the measures against the difference between them. The horizontal black line (line of equality) indicates perfect agreement (difference = 0) between measures. The blue horizontal line shows the arithmetic mean difference across all data points. The dashed lines show the 95 % LoA around the mean difference. The data points do not cluster neatly around the line of equality, demonstrating evidence of heteroskedasticity, that is, the difference between measures varies with the magnitude of the mean in both directions.

As shown by the solid blue line on Fig. 3(b), on average estimates from total household purchases are 129 kcal higher than individual intake (online Supplementary Table 1). However, at lower magnitudes of energy, purchased household energy is lower than energy intake, while at higher magnitudes, purchased household energy is higher than energy intake. Thus, the arithmetic mean difference (online Supplementary Table 1) does not represent well the agreement across the distribution and should be interpreted with caution.

Therefore, as advised by Bland and Altman^(24)^, the data were log-transformed to account for heteroskedasticity (Fig. 3(c)). Here, the mean of log household energy purchased and log individual energy intake is plotted against the difference between log household energy purchased and log individual energy intake. This is shown as a ratio of the difference and can be expressed as a percentage, such that purchases on the log scale represent 66 % of intake on the log scale, though LoA suggest that household purchases for our study population may reasonably be as low as 5 % of energy intake or as high as ten times (987 %) consumed energy levels (online Supplementary Table 1).

Due to the distribution, agreement and LoA are more appropriately shown as regression lines (Fig. 3(d)). The *β*_o_ and *β*_1_ coefficients which make up the regression equations can be found in Table 6. The Bland–Altman plot in Fig. 3(d) shows the agreement for log-transformed variables, with the axes labels back-transformed to aid interpretation. Thus, the x-axis can be interpreted as the mean between household energy purchase and individual energy intake (in kcals) and the y-axis as the difference as a ratio of purchased energy to energy intake. The line of equality (horizontal black line) is now represented by 1 (a 1:1 ratio between measures representing 100 % agreement). Values greater than 1 indicate that purchase estimates are higher than intake, while values lower than 1 indicate purchase estimates are lower than intake.

**Table 6:** Regression coefficients for mean difference and limits of agreement between purchase and intake for energy (kcal)

	Mean difference, (purchase/intake)			Lower limit of agreement		Upper limit of agreement	
	Intercept *b*_*0*_)	Slope (*b1*)	Ratio of difference A = 2000	Intercept (*b*_*0*_)	Ratio of difference A = 2000	Intercept (*b*_*0*_)	Ratio of difference A = 2000
Household purchase – intake							
All households (*n* 686)	−13·25	1·74	0·98	−14·86	0·20	−11·64	4·88
Sensitivity analysis (*n* 562)	−8·19	1·08	1·02	−9·46	0·29	−6·93	3·61
Individual purchase – intake (by household size)							
All households (*n* 686)	−13·60	1·77	0·86	−14·98	0·22	−12·21	3·46
1-person households (*n* 165)	−12·43	1·63	0·96	−13·84	0·23	−11·02	3·94
2-person households (*n* 333)	−13·99	1·84	0·99	−15·29	0·27	−12·69	3·64
3+ persons households (*n* 188)	−12·79	1·67	0·91	−14·24	0·21	−11·34	3·86
(by % shopping with retailer)							
Low–medium loyalty (0–60 %) (*n* 244)	−13·27	1·72	0·82	−14·65	0·21	−11·89	3·25
High loyalty (61–80 %) (*n* 174)	−13·11	1·72	0·96	−14·46	0·25	−11·77	3·70
Very high loyalty (81–100 %) (*n* 268)	−12·49	1·66	1·13	−13·83	0·30	−11·16	4·30

A, average of purchased energy and individual energy intake. For the purposes of comparison, all values are presented for A = 2000 kcal.

Figure 3(d) shows that average agreement between household energy purchased and individual energy intake is near perfect where the mean of values is about 2000 kcal. Yet, the shape of the line indicates that below this magnitude purchased energy is likely to be lower than energy intake, while above this magnitude purchased energy is likely to be higher than energy intake. Taking the intercept and slope of the regression lines (Table 6), it is therefore possible to estimate the expected agreement for a given magnitude. For example, for an average daily intake of 2000 kcal (A), the natural log of A (7·6) is multiplied by the slope, then added to the intercept to give the log of the difference, which is back-transformed to give the ratio of purchase intake. Results suggest that at a mean of 2000 kcal, household purchases under-estimate individual energy intake by just 2 % (about 40 kcal) on average. Yet the wide LoA mean that household energy purchased could be anywhere from just 20 % of energy intake to almost five times higher, demonstrating a lack of confidence in the agreement estimate.

It was observed that a number of customers in our sample had very low daily calorie purchases, which may be influencing our agreement results. Therefore, a sensitivity analysis was conducted excluding customers who purchased less than 500 kcal/d on average (*n* 124). A cut-off of 500 kcal was chosen as this represents a quarter of the daily recommended energy intake for an adult woman, mirroring our upper cut-off of 8000 kcal, which represents four times the recommended intake. Results for the sensitivity analysis (Table 6) show that exclusion of the lowest purchasing customers does not change the mean difference much at an intake of 2000 kcal, yet this time household purchases over-estimate intake by about 2 % on average. Additionally, the slope of the line is reduced and LoA are narrower (as seen on the charts in online Supplementary Fig. 1), indicating closer agreement is observed where the least loyal customers are excluded.

Accounting for household composition, individual-level purchased energy (online Supplementary Fig. 2) under-estimates intake by about 14 % (A = 2000 kcal), yet LoA are narrower than for household purchases (agreement = 86 %, LoA 22 %–343 %). Exploration of subgroups by household size (online Supplementary Fig. 3) show that (for A = 2000 kcal) purchased energy estimates are closest to intake estimates for single-person (agreement = 98 %, LoA 23 % – 393 %) and two-person households (agreement = 99 %, LoA 27 %–365 %), but further for larger households containing three or more persons (agreement = 91 %, LoA 21 %–387 %). For single-person households, household-level and individual-level estimates are equivalent. Yet LoA remain wide, suggesting that purchases are likely to under- and over-estimate intake. There is also an association between agreement of measurements and customer loyalty (online Supplementary Fig. 4). For customers reporting a low-medium loyalty with the retailer (0–60 % of their food shopping), for an average intake of 2000 kcal individual-level energy purchase estimates tend to under-estimate intake, representing 82 % of intake on average (LoA 21 %, 325 %), while in the most loyal customer group (80–100 % of food shopping carried out with the retailer) individual-level purchase estimates over-estimate energy intake (agreement = 113 %, LoA 30 %, 431 %).

Absolute daily purchases at the individual level were also compared with intake for macronutrients and Na (results not presented). To summarise, the nutrients showed similar patterns to those observed for energy; variance in agreement with magnitude of the mean of measures; a tendency for purchases to over-estimate intake at the top end of the distribution and to under-estimate intake at the lower end; and wide LoA. Thus, our results suggest that for all examined nutrients, purchase data provide a poor proxy of individual intake, even when adjusted for household composition.

### Agreement for relative estimates

**Fig. 4:**
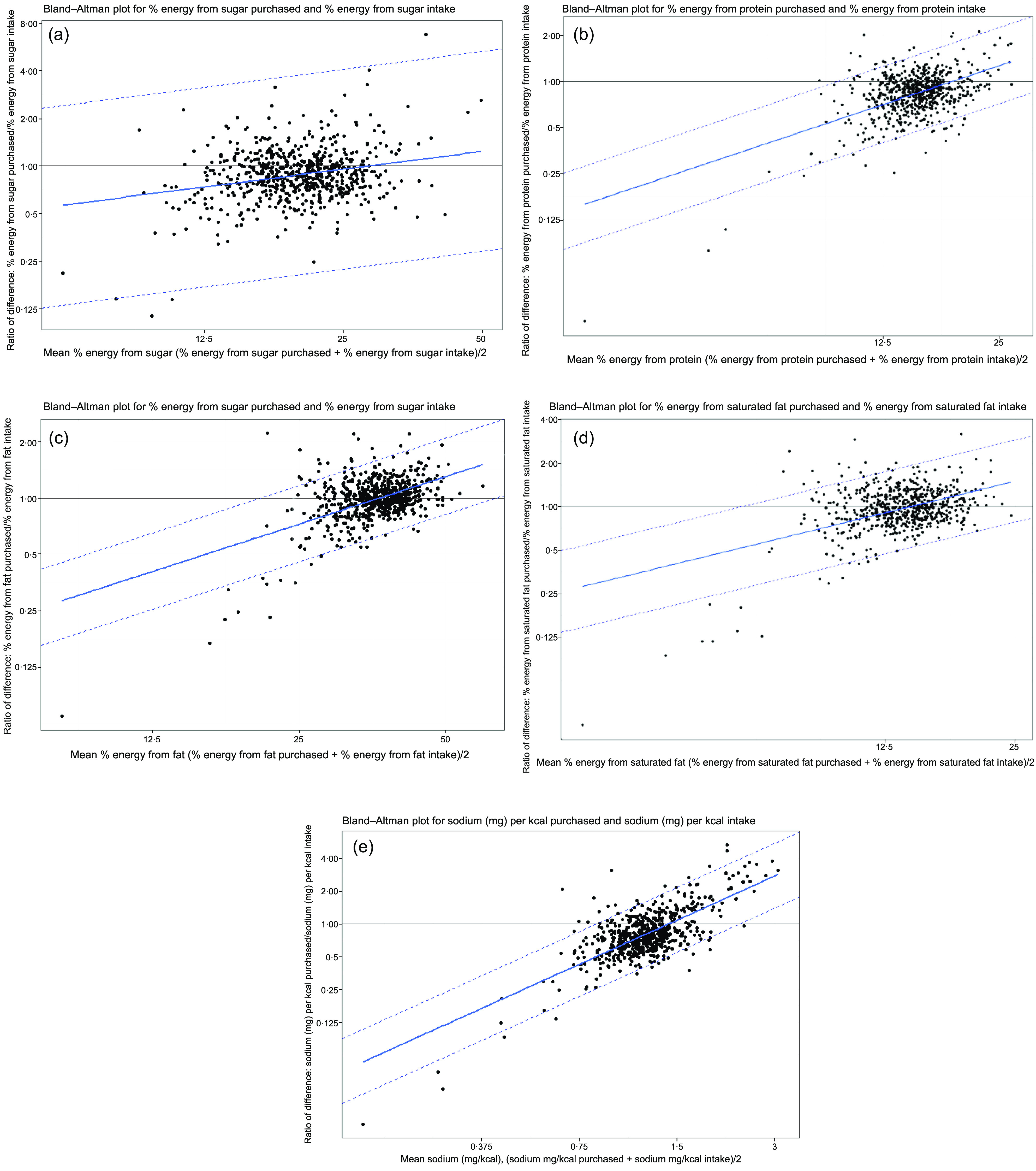
Bland–Altman plots for ratio of relative nutrients purchased (individual level)/relative nutrient intakes, plotted against their average, by nutrient. (a) Sugar, (b) protein, (c) total fat, (d) saturated fat and (e) sodium

Energy-adjusted estimates give an impression of the relative composition of the diet, regardless of volumes purchased or consumed. For relative nutrient estimates, the two measures (purchase and intake) are in much closer agreement than was observed for absolute values (Table 7), as evidenced by a lesser gradient of regression lines and closer LoA (Fig. 4). To aid comparison between nutrients, all results presented in Table 7 are stated for the value of A (average of measures) at which the difference is zero (ratio of difference = 1). For example, where sugar makes up 26·8 % of total energy on average across purchases and intake, the mean of the difference is zero.

**Table 7:** Regression coefficients for difference and limits of agreement for relative purchase and intake for macronutrients and sodium (whole sample, *n* 686)

	Mean difference (purchase/intake)			Lower limit of agreement		Upper limit of agreement	
	Intercept (*b*_*0*_)	Slope (*b1*)	A (for ratio of difference = 1)	Intercept (*b*_*0*_)	Ratio of difference	Intercept (*b*_*0*_)	Ratio of difference
Sugar (% energy)	−1·25	0·38	26·8	−1·77	0·59	−0·74	1·67
Protein (% energy)	−2·47	0·84	18·9	−3·05	0·56	−1·90	1·77
Total fat (% energy)	−3·04	0·84	37·3	−3·51	0·62	−2·57	1·60
Saturated fat (% energy)	−1·93	0·73	14·1	−2·58	0·52	−1·28	1·92
Na (mg/kcal)	−0·46	1·35	1·4	−1·16	0·50	0·23	1·97

A, average of purchased energy and individual energy intake. For the purposes of comparison, all values are presented for the value of A at which the ratio of the difference is 1 (no difference between measures).

While difference and directionality remain related to magnitude, this is to a lesser degree. The closest agreements are observed for sugar (where the ratio of difference = 1, LoA 0·59–1·67) and saturated fat (LoA 0·62–1·60). The greatest difference in agreement is observed for Na, for which where the ratio of difference is 1 and purchases are likely to under-estimate Na/kcal intake by up to a half or over-estimate it by up to two times.

Figure 4(a) shows that purchase estimates of the proportion of total energy to which sugar contributes are typically lower than intake estimates below a mean value of about 25 %. Purchases typically estimate a lower proportion from protein, total fat and saturated fat up to a mean value of about 20 %, 36 % and 12·5 %, respectively. Below a mean of about 1·4 mg/kcal, estimates of Na (mg) per kcal purchased are typically lower than estimated Na (mg) per kcal consumed (Fig. 4(e)).

## Discussion

This paper assesses the agreement between daily intake estimates and daily loyalty card purchase estimates, for energy and five key nutrients (sugar, protein, total fat, saturated fat and Na). Using a unique study dataset, the STRIDE study found agreement to be strongest for smaller households and among the most loyal customers. Absolute purchase values (be they at the household or individual level) were found to be a poor proxy for individual intake. Yet, closer agreement for relative nutrient estimates suggests that purchases represented dietary composition fairly well, making them a good marker of dietary intake pattern. By nutrient, the strongest agreements were found for sugar and saturated fat and for relative values in particular. The STRIDE study contributes to evidence for the validity of purchase records as a proxy for dietary intake. To our knowledge, this is the first study to quantify the statistical agreement and limits to agreement between actual and relative nutrient estimates from automated electronic purchase data and self-reported intake.

Electronically captured purchase records have appeal for their use in population dietary assessment due to their scalability and automated nature. While an obvious limitation is that we do not know exactly what proportion of each customer’s food purchases were carried out with the retailer, we have accounted for some of this variability by asking participants to self-report the retailer’s contribution to their shopping. In addition, it is possible that not all purchases at the retailer may be captured by the data, if an individual forgets to scan their loyalty card for example. That said, automated collection reduces participant and researcher burden greatly increasing the scale and limits the chance of purchases being consciously or subconsciously affected by participation in the study.

Previous comparison studies have described purchase data as a moderately good indicator of intake^(10,12)^ according to correlation of nutrient amounts^(10)^ and association by food category volume and frequency^(12)^. Despite this conclusion, the comparison methods applied were unable to estimate the magnitude of the agreement. As a result, adjustment factors were unavailable to allow for conversion between methods, until now. Using the Bland–Altman method for quantifying statistical agreement, which is considered the gold standard comparator for validation of health research methods^(23,24)^, this study provides a starting point towards developing such adjustment factors.

This study found overall household purchase estimates to be a poor proxy for intake estimates, across all nutrients. LoA were wide, and agreement was also found to be related to magnitude of the mean of estimates. Closest agreement is found at a mean of 2000 kcal/d, suggesting that shopping habits have a greater bearing on agreement than intake. At greater magnitudes, purchase estimates were several times higher than reported intake, even after extrapolation of purchases to the individual level. This is likely to represent people purchasing for others outside the household, for example, for family and friends. At lower magnitudes of the mean, purchase estimates represent just a small fraction of total intake which is likely to be representative of people who eat out a lot or buy the majority of their food from elsewhere. Therefore, whilst purchase records provide poor information on how much an individual is eating, they have potential to reveal information on customer loyalty and household size.

It is likely that over-estimation is due to a combination of large household sizes and inaccuracies in our individual purchase proxy, food waste which may be particularly high among some customers, purchasing for other households (13 % of respondents to the STRIDE baseline questionnaire reported purchasing for others outside of the household as a change in their shopping habits since the COVID-19 pandemic began), and a systematic under-reporting of intake by some participants. While average food waste is estimated to be about 10 %^(28)^, self-reported intake is thought to under-estimate true energy consumption by a similar degree^(5)^. Thus, it is possible that these errors cancel out. Where purchases under-estimate intake, this is most likely due to foods purchased elsewhere and thus not captured by supermarket loyalty card transactions for a single retailer.

Our study is unique in that we attempted to account for household composition to calculate an individual-level purchase estimate for participants. After extrapolation to the individual level, purchase data became more likely to under-estimate intake. Subgroup analysis showed that agreement with individual-level purchase estimates was poorer for larger households. We expect this is due to error built in by the method for allocating nutrients to household members, which increases as the number of people in the household increases. By allocating all nutrients in accordance with age-specific energy intake recommendations, regardless of their food source (e.g. energy derived from alcohol is allocated to children as well as adults), we anticipate greater error for households containing children. The contribution of school meals to children’s diets, differing ratios for dietary requirements by nutrient (e.g. low-salt diets recommended for infants) and unknown genders of other household members could constitute further sources of error. Our finding of poor agreement between purchases and intake for absolute nutrient values is not unexpected given the data only represents purchases from a single retailer. Indeed, our findings mirrored those from Vepsalainen et al.^(12)^, in that we found closer agreement for smaller households and more loyal customers (according to self-reported proportion of shopping with the study retailer).

Differences in agreement by nutrient were observed, in line with previous findings which reported strongest relationships for total fat and saturated fat^(10,14)^ and variation in concurrence by food group^(12)^. Agreement for saturated fat was slightly lower, which may be due to a higher tendency to purchase high-saturated fat treat items elsewhere. For example, crisps and sweet treats are often consumed on the go, purchased at cafes, petrol stations and from vending machines^(29)^. The lowest agreement was observed for Na, which may reflect that other food sources (e.g. out of home) contribute a relatively higher proportion of salt to the diet (restaurant and takeaway meals have been found to contain higher levels of salt than home-cooked or ready meal equivalents^(30)^). Purchase data may poorly account for table salt added to food at home, which tends to be purchased in large quantities but relatively infrequently. It is also likely that the time period covered by purchase data influences the degree of agreement with consumption of salt and other store-cupboard items. This theory is supported by findings by Vepsalainen et al.^(12)^, who reported weak associations for vegetable oil, as well as a general trend for stronger associations when comparing intake with 12-month purchase data compared with just 1 month. Exploration of the timescale required of purchase records to capture habitual diet is therefore warranted.

Adjusting nutrients for total energy allows for comparison of relative dietary composition, rather than absolute nutrient quantities. As expected, relative nutrient purchases showed a higher agreement with intake, particularly for total fat and sugar, similar to the observations made by Eyles et al.^(10)^ who also reported a high correlation for total fat. Furthermore, the relationship with customer loyalty across relative measures was less apparent than for absolute measures. This indicates that while purchases from a single retailer tend to under-estimate nutrient intake in absolute terms, they are relatively reflective of overall dietary choices. Proportion of purchased energy from macronutrients could therefore provide a useful surrogate marker for dietary quality^(13)^. This supports the validity of transaction data in dietary patterns research^(17)^ and for ecological research applications, such as evaluating policy impacts^(31)^ and identifying population-level trends such as the increasing popularity of plant-based protein sources^(32)^.

A limitation of this study is that, due to its prospective nature, it was not possible to sample customers based on their loyalty to the retailer during the study period. While we made an attempt to account for customer loyalty by selecting customers who purchased regularly with the supermarket during the year prior to recruitment, it was apparent that previous loyalty did not reflect customer purchasing behaviours during the study period. This observation may have been unique due to the circumstances of the COVID-19 pandemic, which saw many customers switching to different retailers due to the proximity of stores or availability of online delivery slots. Studies using retrospective sampling approaches should therefore account for customer loyalty when selecting study participants to improve representation of intake. If customer cohorts are to be followed over time, characterisation of customer loyalty and re-sampling are likely to be beneficial to ensure the sample remains representative of loyal customers.

Customer loyalty is revealed as an important influencer of agreement between dietary estimates from purchase records from a single and self-report methods. The importance of loyalty could be mitigated if purchase data from multiple sources could be combined for individual customers, giving a more complete picture of overall food purchases. However, ability to link purchase data sources is limited by commercial sensitivity and competition law as well as the ability to identify individuals within anonymised transaction records. Exploration of informed consent models are required to facilitate novel data linkage and improve integration with other research assets such as cohort studies.

A further limitation is that our study population appears to be relatively affluent according to the characteristics of their home neighbourhoods, although we did not assess participant income directly. Affluence is commonly found to be related to dietary and purchase decisions, including where one chooses to shop, how often they eat out and the types of products they buy. It may reasonably be hypothesised, for example, that agreement may be poorer among less affluent communities who are more likely to shop around to find the best prices. It is therefore unclear how well our findings would represent agreement between purchases and intake for less affluent customers of a discount grocery chain, for example. Furthermore, while participant demographic characteristics differed little between sign-up and completion, it is impossible to know how well our sample reflects the overall customer base as this information was not shared by the retailer. Good sample characterisation is therefore important when working with loyalty card data. However, large customer samples afford the capacity for adjustment for improved national representation.

A strength of this study is the use of a bespoke product nutrient composition database which combines product-level composition data from the back of pack nutrition label with generic food composition data. By using actual product composition information where possible, the accuracy of nutrient estimates from purchase data in maximised. Indeed, it may be true that for some foods (particularly for composite dishes, for which there may be just one option available in generic food tables compared with many different products on the retailer’s shelves), nutrient estimates at the product level are likely to more accurate than for intake estimates as they enable accounting for brand-level differences. Furthermore, the database used gave extremely good coverage of product nutrient data across all food categories, rather than being restricted to just the most commonly purchased foods as in the study by Eyles et al.^(10)^.

### Future research avenues

The STRIDE study provides a rich dataset which will enable further investigation of differences in agreement according to customer demographic characteristics (such as age, gender and BMI), by season and by geography (according to geodemographic classification and area-level deprivation indices).

Future work should also explore what volume of transaction records are most suitable for assessing habitual diets, taking into account their ability to capture less frequently purchased bulk or store-cupboard items, and seasonal dietary patterns. Additionally, methods are required to estimate household size and composition, as well as customer loyalty in the absence of survey data.

### Conclusion

This study demonstrates progress towards the generation of adjustment factors for extrapolation of household purchase estimates to individual intake estimates. In this setting, where it was not possible to restrict the customer sample to only shoppers who purchase most of their food from the study supermarket, we found poor agreement between absolute nutrient measures from purchase data and self-reported intake. Agreement was strongest for single-person households, loyal customers, energy, total fat and sugar, providing evidence that customer sampling is an important consideration for studies using supermarket transaction data. Relative (energy-adjusted) nutrient estimates provide a good indicator of dietary composition (which appears to be unrelated to customer loyalty), which may be beneficial for ecological studies, identification of intervention target groups, and monitoring of dietary patterns and quality with applicability for policy evaluation.

## Supplementary material

For supplementary material accompanying this paper visit https://doi.org/10.1017/S1368980023001842
